# Impact of Gut Microbiota on Host Glycemic Control

**DOI:** 10.3389/fendo.2019.00029

**Published:** 2019-01-30

**Authors:** Céline Gérard, Hubert Vidal

**Affiliations:** Univ Lyon, CarMeN Laboratory, INSERM U1060, INRA U1397, INSA Lyon, Université Claude Bernard Lyon1, Oullins, France

**Keywords:** obesity, diabetes, microbiota, glucose metabolism, probiotics

## Abstract

Given that obesity and associated disorder type II diabetes mellitus have reached epidemic proportions worldwide, the development of efficient prevention and therapeutic interventions is a global public health interest. There is now a large body of evidence suggesting that the micro-organisms colonizing the human gut, known as gut microbiota, play a central role in human physiology and metabolism. Understanding how gut microbiota affects and regulates key metabolic functions such as glucose regulation and insulin resistance is an important health issue. The present review summarizes recent advances in our understanding of how gut bacterial species interfere with host metabolic phenotype. We will examine key biological molecular mechanisms underlying the impact of gut microbiota on host glycemic control including: incretin secretion, short-chain fatty acid production, bile acid metabolism, and adipose tissue regulation. We will highlight how prebiotic/probiotic interventions affect these bacterial processes and are now considered as promising approaches to treat obese and diabetic patients.

## Overview

Obesity is a chronic, complex, and multifactorial disease representing the fifth leading cause of death in the world and accounting for almost 3.4 million deaths each year (1).

In adults, obesity is characterized by an excessive fat accumulation and is defined conventionally as a body mass index equal or higher than 30 kg/m^2^. Epidemiological studies indicate that more than 2.1 billion people are currently overweight or obese worldwide. If current trends continue, estimations show that 38% of the world's adult population will be overweight and another 20% will be obese by the year 2030 (1, 2). Obese state is associated with multiple severe comorbidities and complications (3). In the same way as obesity, the prevalence of diabetes has increased tremendously around the world and is becoming a leading cause of death in many countries. The International Diabetes Federation (IDF) Diabetes Atlas, 8th Edition, 2017 highlights some alarming statistics: some 425 million people worldwide, or 8.8% of adults 20–79 years, are estimated to have diabetes. If these trends continue, the number of people 20–79 years with diabetes worldwide will reach 629 million by 2045 (4). Type 2 diabetes mellitus (T2DM) accounts for more than 90% of the cases. Approximately 5.0 million deaths worldwide were attributable to diabetes in 2017, which is equivalent to one death every 8 s (4). Diabetes results in exceptional healthcare costs; it is estimated that more than 700 billion US dollars are spent yearly by people with diabetes, which corresponds to one for every eight dollars spent on healthcare (5, 6). Although diabetes mainly occurs in people aged over 40 years old, it is also becoming increasingly prevalent in younger age groups (7).

T2DM is diagnosed by the presence of elevated blood glucose levels (hyperglycemia), which is the hallmark of the disease. Hyperglycemia is due to concomitant insulin resistance and insufficient insulin secretion due to beta-cell function impairment over time, resulting in failure to control blood glucose levels. Even if the precise mechanisms by which obesity leads to insulin resistance affecting skeletal muscle and liver, but also adipose tissue and brain, are not fully understood, epidemiological studies have established that T2DM is the result of complex gene-environment interactions. Excess caloric intake and reduced energy expenditure are important predictors of obesity and T2DM. Recent experimental and clinical evidence suggests that another key endogenous factor may be considered as a critical host metabolism regulator: the gut microbiota. Although still debated, it is estimated that up to 100 trillion micro-organisms reside throughout our body and that the collective gene set of our gut microbes is about 150 times larger than our own human genome (8, 9). Microbiota is now recognized as a real functional “organ” due to its immense impact on human health and has become the subject of intensive research over recent years. The vast majority of microbes reside in the intestinal tract, where they influence host physiology by playing fundamentally important roles in digestion, nutrition, immune regulation, and metabolism. Gut microbiota composition and activity can fluctuate over time and depend on different factors including genetics, sex, age, health status, and drug/antibiotic consumption (10). Over the last decade, a large number of publications have reported a prominent role of microbiota in metabolic diseases. Notably, accumulated evidence suggests an association between a dysregulated gut microbiome and obesity, glycemic control impairment, and therefore T2DM pathophysiology (11, 12).

## Obesity, Diabetes, and Dysbiosis

The preservation a normal and healthy gut microbiota plays a critical role in maintaining good health. *Bacteroidetes* and *Firmicutes*, including species of the *Ruminococcus, Lactobacillus*, and *Clostridium* genera, constitute over 90% of the known phylogenetic categories and dominate the healthy intestinal microbiota (13). To a lesser extent, species belonging to other phyla such as *Actinobacteria, Verrucomicrobia*, and *Fusobacteria* are also present. Alterations of both composition and function of the microbiota, termed dysbiosis, are common features of several pathologies including metabolic diseases such as obesity and T2DM.

A number of preclinical and clinical studies have attempted to describe the differences between gut microbiota in obese compared to lean individuals and have reported that obesity condition is related to lower microbial diversity and taxon depletion (14, 15).

Early obesity-microbiota studies report that an increase of body weight is associated with a microbiota shift identified by a change in the *Bacteroidetes/Firmicutes* ratio with a larger proportion of *Firmicutes* and a decline in *Bacteroidetes* populations. An increased ratio of *Firmicutes* to *Bacteroidetes* has also been described in a model of mice genetically predisposed to obesity (ob/ob), with a 50% reduction in the abundance of *Bacteroidetes*, and a proportional increase in *Firmicutes* compared to their lean siblings (16). Importantly, controversial data have been reported in more recent studies. Schwiertz et al. observed opposite results and determined lower ratios of *Firmicutes* to *Bacteroidetes* in overweight human adults compared to lean controls (17). Also, in other studies, authors found no proof of the association between the proportion of *Bacteroidetes* and *Firmicutes* and human obesity (18, 19). Some of the variability of results reported between studies might be due to differences in laboratory protocols, study design or different methodology used over time. Moreover, it is noteworthy to mention that the measure of the *Bacteroidetes/Firmicutes* ratio is a rough method to characterize the microbiota. More standardized and accurate procedures are needed to compare studies from different laboratories as well as a more taxonomically detailed description than phylum level changes (20).

Dysbiosis has been linked to important metabolic consequences and profound deregulations: a higher expression of microbial genes that encode enzymes related to carbohydrate metabolism and a tendency to an overgrowth of bacteria more efficient at extracting energy from food, inducing excessive fat accumulation (21).

Although T2DM is generally considered as an attribute to obesity, recent metagenomics approaches have helped define the specific composition of fecal microbiota in T2DM patients. Interestingly, some studies have correlated glycemic control impairment and insulin resistance to specific gut microbiota composition. In 2012, deep shotgun sequencing of the gut microbial DNA from 345 Chinese individuals showed that patients with T2DM were characterized by a moderate degree of gut microbial dysbiosis, a decrease in the abundance of some universal butyrate-producing bacteria, and an increase in various opportunistic pathogens (22). Later, Karlsson et al. used shotgun sequencing to characterize the fecal metagenome of 145 European women with normal, impaired or diabetic glucose control (20). Authors highlighted compositional and functional modifications in the metagenomes of T2DM patients, and proposed a mathematical model based on metagenomics signatures that identified T2DM with high accuracy. However, they concluded that metagenomics predictive tools for T2DM should be specific for the age and geographical location of the populations studied (20). Very recently, prediabetes state (defined as fasting plasma glucose of 6.1–7.0 mmol/l) has also been associated with aberrant gut microbiota profiles (23). This case-control study including 134 Danish adults with prediabetes and 134 healthy individuals with normal glycemic control showed that the abundance of five bacterial genera and 36 operational taxonomic units (OTUs) were altered in individuals with prediabetes compared to those with normal glucose regulation. Notably, mucin-degrading bacterium *Akkermansia muciniphila* was found at lower abundance in microbiota of individuals with prediabetes (23). These findings suggest that gut microbial alterations may represent a disease signature and a potential tool to distinguish individuals presenting a precursor state of T2DM.

Collectively, these data clearly suggest that the composition/function of gut microbiota may contribute to host glycemic regulation and insulin sensitivity. Furthermore, antidiabetic drugs liraglutide and metformin have been recently shown to significantly lower body weight and improve glucose metabolism while modifying considerably the composition of gut microbiota (24, 25). Notably, liraglutide decreased obesity-related microbial phenotypes and increased lean-related phenotypes (24). In a comparable approach, several preclinical and clinical studies highlighted that metformin modifies the intestinal microbiota composition by inducing the growth of several bacteria, such as *Akkermansia muciniphila* (26, 27). Moreover, fecal transfer to germ-free mice improved glucose homeostasis in recipients of samples from patients who received metformin (28). These last data suggest that gut microbiota is involved in the beneficial glucose-lowering effects of antidiabetic agents and confirm that gut microbiota is a promising therapeutic target in T2DM and the glycemic control impairment context. Importantly, since these antidiabetic drugs have a major impact on gut microbiota composition, they might partly explain inconsistent and conflicting results observed among studies investigating gut microbiota composition in obese and T2DM patients. This further highlights the necessity to design and compare standardized studies and to take into account the consumption of these drugs before establishing any causal relationships.

**Figure 1:**
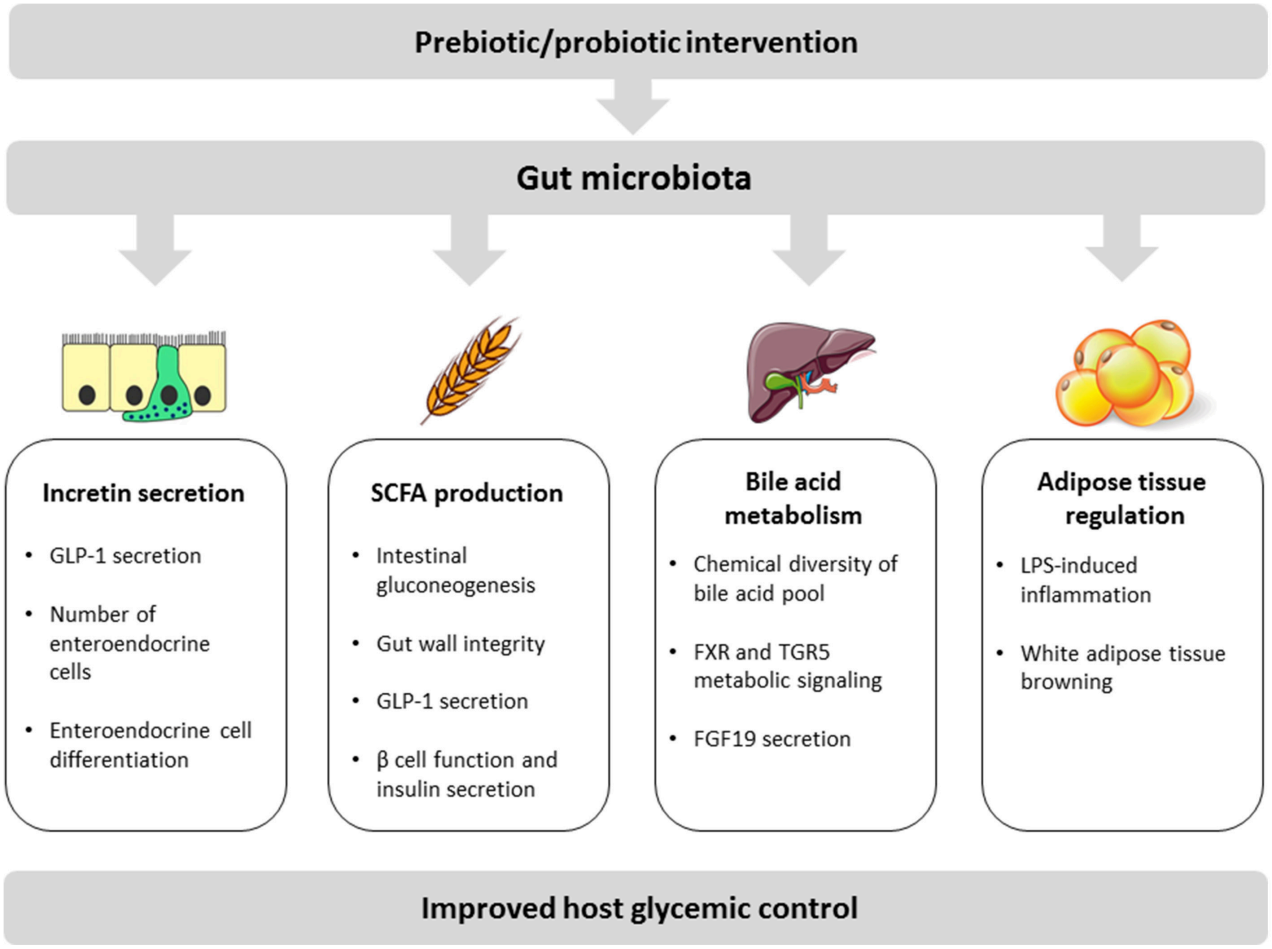
Schematic view of key mechanisms linking the gut microbiota with host glycemic regulation. These mechanisms include (1) increase of incretin secretion either through direct induction of GLP-1 production or via an increase of the number/differentiation of enteroendocrine cells; (2) bacterial production of SCFA with beneficial impacts on intestinal gluconeogenesis, gut wall integrity, incretin secretion, and pancreatic functions; (3) bacterial metabolism of bile acids contributing to bile acid pool diversity and inducing local and peripheral signaling effects including via FGF19 production in the gut; (4) adipose tissue regulation mainly through modulation of LPS-mediated inflammation and induction of white adipose tissue browning.

The main proposed molecular mechanisms by which the gut microbiota modulates and interferes with host glycemic control are discussed below and include: modulation of incretin secretion, short chain fatty acid production, bile acid transformation, and regulation of adipose tissue inflammation and function (summarized in Figure 1).

## Intestinal Incretin Secretion

Incretin hormones, mainly represented by glucagon-like peptide-1 (GLP-1) and gastric inhibitory polypeptide (GIP), are gut peptides released from enteroendocrine cells. They are secreted into blood stream and rapidly stimulate insulin secretion from beta cells in response to nutrients in order to control meal-related glycemic excursions (29). Oral glucose load induces a much greater insulin response than that of an isoglycemic intravenous glucose infusion. This is known as the “incretin effect,” which is responsible for 50–60% of postprandial insulin secretion in healthy individuals. In addition to its insulinotropic action, GLP-1 exerts numerous other functions related to metabolic health. Most relevant are: a reduction in appetite, pancreatic beta cells proliferation, food-driven gastrointestinal motility, and gastric emptying which offer unique benefits in terms of weight loss in treatment of obesity and T2DM (30). Importantly, incretin peptides are rapidly degraded and inactivated by endogenous protease, dipeptidyl-peptidase 4, resulting in very short plasma half-lives for the active forms of GIP, and GLP-1. Incretin secretion is markedly impaired in individuals with obesity and T2DM compared with healthy individuals. A large Danish study, published in 2015 and involving 1,462 individuals, showed a reduced GLP-1 response to an oral glucose tolerance test (OGTT) in prediabetes, diagnosed T2DM, and obese individuals compared to normal glucose-tolerant individuals and normal weight individuals, respectively (31). The results of this study indicate that GLP-1 secretion impairment occurs prior the establishment of obesity and T2DM, suggesting that altered incretin response may be a signature for early detection and prevention of diabetes development. Interestingly, both men and woman with prediabetes or T2DM presented up to 21% reduced GLP-1 response compared with normal glucose-tolerant individuals (31, 32). In this context, two forms of incretin-based therapeutic approaches have therefore emerged to treat diabetic patients: GLP-1R agonists to mimic and potentiate the action of endogenous GLP-1 and dipeptidyl-peptidase four inhibitors to prevent the degradation of GLP-1 and GIP (33).

Several studies have attempted to decipher how gut microbiota regulates and interferes with incretion secretion. Notably, authors have showed that the fermentation action of gut microbiota in the colon could impact the number of enteroendocrine cells. The addition of non-digestible carbohydrates, such as oligofructose, in the diet has been shown to improve glucose tolerance, insulin response, and to reduce food intake in mice and humans. These beneficial metabolic effects have been linked to higher plasma GLP-1 levels (34–36). To confirm the association between the beneficial impact of dietary non-digestible carbohydrate ingestion and increased GLP-1 secretion, Cani et al. have demonstrated that the effects of oligofructose were abolished when administered to GLP-1 receptor knock-out mice or mice chronically treated with GLP-1 receptor antagonist (36). The increase of GLP-1 secretion following non-digestible carbohydrate intake was further delineated in a study conducted in Wistar rats and attributed to an induction of L-cell differentiation in the colon via the up-regulation of two differentiation factors: Neurogenin 3 and NeuroD. Immunohistochemistry analyses revealed that the number of enteroendocrine L-cells was doubled in the proximal colon of rats treated with oligofructose, contributing to a higher endogenous GLP-1 production (37).

Some bacteria can directly regulate incretin secretion by the metabolic compounds they produce. Pichette et al. have identified that hydrogen sulfide (H_2_S), a bioactive gas metabolite abundantly produced in the colon by sulfate-reducing bacteria, can directly stimulate intestinal GLP-1 response (38). Authors showed that H_2_S donors, NaHS, and GYY4137, efficiently increased GLP-1 secretion in an *in vitro* model of L-cells through p38 mitogen-activated protein kinase pathway. Enrichment of mice gut microbiota with sulfate-reducing bacteria is possible via supplementation of food with prebiotic chondroitin sulfate. After 4 weeks of prebiotic intake, these mice were characterized by improved GLP-1 and insulin response, improved glycemic control, and lower food consumption (38). However, it has also been demonstrated that H_2_S presented potent inhibitory effects on GLP-1 release in response to bile acid receptor TGR5 in an *in vitro* model (39). Further work is therefore required to disentangle the exact role of sulfur-reducing bacteria of the gut microbiota and sulfate prebiotics on incretin system and glucose metabolism. In a same approach, it has been shown that indole, a metabolite produced from the dissimilation of tryptophan by gut microbiota, is able to modulate the secretion of GLP-1 from immortalized and primary mouse colonic enteroendocrine L cells (40). Authors have demonstrated that indole enhanced GLP-1 release during short periods of exposure, but reduced its secretion after longer treatment, identifying indole as a potent signaling molecule by which gut microbiota may interact with enteroendocrine cells and alter host glycemic control.

Other interesting mechanisms of action by which gut microbiota interferes with the incretin axis have been recently described. In a study conducted by Yang et al. authors have suggested that gut microbiota acted on myenteric neural cells where it induces a decrease of GLP-1 receptor expression to promote gastrointestinal motility (41). In another study published in 2017, Grasset et al. describe a brand new mechanism by which gut microbiota can interfere with incretin resistance (42). They indeed identified a specific set of ileum bacteria impairing the GLP-1-activated gut-brain axis, leading to a state of GLP-1 resistance. Their results indicate that insulin secretion and gastric emptying require functional GLP-1 receptor and neuronal nitric oxide synthase in the enteric nervous system within a eubiotic gut microbiota environment, opening a promising novel strategy to potentiate GLP-1-based therapies (42).

Several probiotic interventions have been reported to ameliorate glucose control and attenuate T2DM state by increasing incretin secretion. Daily treatments with specific bacterial strains in different animal models of T2DM have been shown to attenuate symptoms of diabetes, including postprandial blood glucose and insulin resistance via an increase of intestinal GLP-1 levels (43, 44). Although the precise mechanisms of action of such probiotics are not known, the efficacy to induce incretin secretion of several bacterial strains has been confirmed in humans. In a prospective randomized trial including 21 glucose-tolerant individuals, daily intake of *Lactobacillus reuteri* SD5865 for 4 weeks enhanced glucose-stimulated GLP-1 response by 76% compared with control group. This enhanced GLP-1 secretion was associated with an increase of insulin and C-peptide concentrations of 49 and 55%, respectively (45). Interestingly, a new incretin-based probiotic approach has recently emerged; a recombinant microbial delivery vector for long-acting GLP-1 analog has been designed and tested in diet-induced obese rodents (46). Animals receiving *Lactobacillus paracasei* NFBC 338 transformed to express the GLP-1 analog showed enhanced insulin secretion after glucose stimulation and improved glucose metabolism compared to the animals in the control group receiving the isogenic control microbe which solely harbored the pNZ44 plasmid. This study suggests that recombinant incretin-secreting microbes may offer a novel therapeutic strategy to manage host glucose control.

## Short-Chain Fatty Acid Production

Short chain fatty acids (SCFA) are organic fatty acids containing two to six atoms of carbon. They are produced in the colon and the caecum of the host following the anaerobic fermentation of non-digestible dietary fibers by saccarolytic bacteria (47). Acetate, propionate, and butyrate represent 95% of SCFA, and are among the most abundant microbiota-derived compounds. They are detected in millimolar concentrations (around 60, 20, and 20 mM, respectively) in the gut lumen and absorbed via the colonic epithelium to achieve blood concentrations of up to 150 μM for acetate, whereas propionate and butyrate can only reach 1–10 μM (48, 49). Once in the circulation, they can be used for *de novo* lipid or glucose synthesis (50). Short chain fatty acids are a notable example of mutualistic relationship between the gut microbiota and the host; the host diet provides non-digestible dietary fibers to promote bacterial growth and, in turn, the gut bacteria generate SCFA that can be used as substrates by the host.

In addition to being an important source of energy for the host, SCFA contribute to many biological pathways including amelioration of the host metabolic health. Notably, they are involved in intestinal health by impacting colonocytes proliferation, differentiation, and inflammation (51, 52). Moreover, they have been described to improve host glycemic control (53). In line with these key metabolic roles, deficiency in SCFA synthesis has been associated with diabetes pathophysiology. Using 784 available human gut metagenomes, Forslund et al. reported a unified profile of gut microbiome shifts in T2DM patients with a notable depletion of butyrate-producing taxa (25), consistent with previous observations (20, 22). It is noteworthy that hundreds of gut bacterial species across many taxa present the genetic capacity for fermenting carbohydrates and are able to produce SCFA (54). Nevertheless, a recent randomized clinical study using fecal shotgun metagenomics demonstrated that only a small number of SCFA-producing strains (15 strains from three different phyla) were promoted by dietary fiber supplementation and were able to benefit from this new dietary resource (55). Most other potential SCFA producers remained unchanged in patients with T2DM. These 15 strains are therefore the potential key players in the mutualistic relationship between the intestinal microbiota and the host. These data suggest that targeted restoration and promotion of these specific SCFA producers may represent a novel ecological approach for managing T2DM.

At a local level, propionate and butyrate have been described to stimulate intestinal gluconeogenesis, via different mechanisms (56). Intestinal gluconeogenesis is a mucosal process exerting anti-diabetic and anti-obesity effects via the activation of gut-brain neural circuits and therefore improving glucose and energy homeostasis (57). Notably, butyrate induces the expression of genes involved in intestinal gluconeogenesis through a cAMP-dependent mechanism, while propionate, itself a substrate of intestinal gluconeogenesis, activates gene expression via a gut-brain neural circuit (56). Authors further demonstrated that metabolic benefits on glucose homeostasis induced by SCFA or dietary fiber in mice are not observed in mice deficient for intestinal gluconeogenesis, despite similar modifications in gut microbiota composition. The regulation of intestinal gluconeogenesis is therefore necessary for the metabolic benefits associated with SCFA and soluble fibers. It is noteworthy that although research has mostly focused on the production of SCFA, bacterial fermentation of dietary fiber also generates high concentration of succinate. Importantly, dietary succinate has also been identified as a substrate for intestinal gluconeogenesis (58, 59). Additionally, butyrate has been linked to enhanced gut wall integrity (60, 61). Xu et al. have provided evidence that oral butyrate administration significantly lowered plasma levels of HbA1c, inflammatory cytokines, and lipopolysaccharide (LPS) in db/db mice (62). They further showed that local inflammatory cell infiltration was reduced while gut integrity and intercellular adhesion molecules were increased after butyrate treatment. These findings suggest that butyrate could ameliorate gut-leak and diabetic-endotoxemia in db/db mice by preserving gut epithelial barrier integrity and may therefore ameliorate LPS-induced insulin resistance.

The discovery of SCFA receptors in many different tissues has highlighted SCFA as important signaling molecules involved in the metabolic crosstalk between the gut and peripheral tissues. Notably, two G protein-coupled receptors have been described to be activated by SCFA and identified as modulators of microbiota-host interaction: GPR41 and GPR43, also known as the free fatty acid receptors FFAR3 and FFAR2, respectively (63). They have been detected in the gut, in the sympathetic nervous system, in the liver, white adipose tissue, skeletal muscle, pancreas, and immune tissues (64).

GPR43 has been identified to link the metabolic activity of the gut microbiota with host body energy homoeostasis. GPR43-deficient mice have been shown to be obese on a normal diet, whereas mice overexpressing GPR43 specifically in adipose tissue remain lean even when fed a high-fat diet (65). Raised under germ-free conditions or after treatment with antibiotics, both types of mice have a normal phenotype, demonstrating the importance of microbial metabolism in forming ligands for GPR43 signaling. However, there is some controversy over the exact role of GPR43 in the regulation of energy balance *in vivo*. Inconsistently, in another study, high fat diet-fed GPR43—deficient mice were shown to have lower body fat mass associated with improved glucose control and insulin sensitivity as well as increased energy expenditure compared to normal diet-fed mice (66). Although SCFA/GPR43 axis seems to play an important function in obesity, weight gain, and adiposity, its exact role is still far from clear and needs to be clarified (67). Importantly, SCFA have been described to stimulate the secretion of a number of gut peptide hormones, including the incretin GLP-1 controlling insulin release and appetite, mainly via GPR43 (68). Mice lacking GPR41 or GPR43 exhibited reduced SCFA-triggered GLP-1 secretion *in vitro* and *in vivo* accompanied by the deterioration of glucose metabolism. Consistent with these observations, some studies have showed that propionate was able to stimulate GLP-1 secretion *in vivo* via GPR43 in Wistar rats and C57BL6 mice (69). Infusion of propionate in the colon of rats enhanced GLP-1 levels both in jugular vein plasma and in portal vein plasma of these animals. However, propionate did not significantly stimulate gut hormone release in GPR43 knockout mice demonstrating the implication of this receptor in incretin secretion. A supplementation with probiotic *VSL#3* has been shown to promote the release of the incretin GLP-1, resulting in reduced food intake and improved glucose tolerance via an increase in the levels of a SCFA butyrate in several mouse models (70). Additional beneficial roles of SCFA receptors have more recently emerged in pancreatic beta cells function and physiology (71). Authors have demonstrated that specific agonist of GPR43 potentiates insulin secretion in a GPR43-Gαq and phospholipase C-dependent manner, identifying GPR43 as a potential target for therapeutic intervention in T2DM context (72).

A large number of studies have highlighted beneficial anti-diabetic effects of supplementation with SCFA or dietary fibers, which increase SCFA production, or bacterial colonization. For example, sodium butyrate supplementation resulted in reduced fasting glucose and greater insulin sensitivity in a model of mice fed a high fat diet (73). In the same manner, Zhang et al. have demonstrated that 1% butyrate supplementation in the drinking water of high-fat diet fed mice lowered serum insulin and fasting glucose levels compared with high-fat diet controls (74). Moreover, supplementation with prebiotic oligofructose in high fat fed mice induced a remarkable increase of beneficial *Bifidobacterium* species that was correlated with improved glucose tolerance and glucose-induced insulin secretion (37). In humans, improved postprandial glucose response and higher abundance of butyrate-producing bacteria were observed after a treatment of 3 months with a prebiotic mixture of inulin and oligofructose in obese women (64, 75). Very recently, a double-blind randomized clinical trial enrolling 14 overweight to obese men showed that an acute ingestion of a high-fat milkshake containing prebiotic inulin leads to lower postprandial plasma glucose and insulin levels compared with the group receiving the high-fat milkshake with digestible carbohydrates (maltodextrin) (76). Authors further showed that inulin was fermented into SCFA as revealed by higher plasma acetate concentrations compared with the digestible carbohydrates group, suggesting that substituting digestible carbohydrates with the fermentable inulin may benefit host metabolism and glycemic response.

## Bile Acids Metabolism

Bile acids are potent digestive surfactants that aid in the digestion and absorption of lipids and fat-soluble vitamins by acting as emulsifiers.

Primary bile acids are synthesized exclusively in hepatocytes from cholesterol after a series of enzymatic reactions. They are thereafter conjugated primarily to glycine and taurine, stored, and concentrated in the gallbladder, and secreted into the intestinal lumen upon food intake. Most of the bile acids are reabsorbed from the small intestine by active transport in the terminal ileum and returned to the liver via the portal circulation. This process, known as enterohepatic recirculation, allows efficient bile acid recycling and is repeated multiple times each day. Only ~5% of bile acids are not reabsorbed and are eliminated in the feces. The size of the bile acid pool is tightly controlled, and bile acids regulate their own hepatic synthesis via a negative feedback regulatory mechanism (77).

Feedback regulation and downregulation of specific enzymes expression involve direct interaction between bile acids and farnesoid x receptor (FXR) in hepatocytes. Alternatively, the interaction bile acids/FXR in ileal enterocytes induces the expression of fibroblast growth factor (FGF19, designated as FGF15 in mice) which enters the circulation and further inhibits bile acid synthesis through activation of hepatic fibroblast growth factor receptor 4 (FGFR4) (78).

For in-depth reviews on bile acid synthesis and feedback regulation, the reader is referred to following reviews: (79, 80). There is increasing evidence that bile acids are not only simple surfactant molecules that facilitate digestion and promote lipid absorption in the intestine. Over the last decade, it has become clear that they are also potent signaling molecules, mainly via interaction with nuclear receptor FXR and membrane G protein-coupled receptor TGR5 (also known as GPBAR-1 or GPCR19), having substantial impact on health and disease including obesity and T2DM. Hepatic overexpression of CYP7A1, the rate-limiting enzyme in the bile acid biosynthetic pathway, in obese mice has been shown to cause weight reduction and protection from glucose intolerance and insulin resistance in addition to an expansion of the bile acid pool (81). These data suggest that manipulation of the bile acid pool may represent a potential therapeutic strategy for managing metabolic diseases such as obesity and diabetes.

Bile acids and gut microbiota are mutually linked and closely affect each other. Bile acids that escape from ileal reabsorption enter the colon where they are transformed into secondary bile acids by the resident gut microbiota (82). Enzymatic activity of several bacteria species leads to the deconjugation (carried out by the enzyme bile salt hydrolase), dehydrogenation, and dehydroxylation of primary bile acids to form secondary bile acids, modifying the chemical diversity, and contributing to heterogeneity of bile acid pool (83).

Several studies have highlighted the importance of bile acid modification genes in the gut microbiome in host metabolic status by quantifying the abundance of bile salt hydrogenase (BSH) gene, 7 alpha-dehydroxylase (ADH) gene, and 7-alpha hydroxysteroid dehydrogenase (HSDH) gene in fecal samples from T2DM patients. They showed that gut microbiota of T2DM patients was characterized by a significant reduction in firmicute-derived BSH. Reduction of ADH and HSDH genes was also found in T2DM patients relative to healthy controls (84).

Probiotic strategy can rapidly influence bile acid metabolism and reverse deficiencies in BSH gene and may prove useful in chronic metabolic disorders. A daily supplementation with BSH-active *Lactobacillus reuteri* NCIMB 30242 daily was shown to increase circulating bile acids by >2-fold as a result of enhanced intestinal bile acid deconjugation, which was associated with an increase of FGF19 secretion (85). Interestingly, recent data suggest that the differential therapeutic responses to the antidiabetic drug acarbose are related to composition of the gut microbiota prior to treatment through distinct abilities of the microbial communities to metabolize bile acids (86). Patients having a gut microbiota enriched with *Bacteroides* presented more modifications in plasma bile acids as well as better amelioration of their metabolic parameters after acarbose treatment compared to individuals with gut microbiota dominated by *Prevotella*. These findings suggest that gut microbiota composition may provide a predictive tool to stratify patients according to their probability to respond to antidiabetic drugs and select appropriate and individualized medication strategy.

Gut microbiota should therefore be considered as a pivotal factor in host bile homeostasis and any modification of microbial community might perturb host bile acid profile and alter host physiological functions. This is clearly demonstrated by the important alteration of bile acid pool observed in germ free mice compared to conventional mice (83, 87). Studies further showed that the gut microbiota not only impacts secondary bile acid pool but also inhibits hepatic bile acid synthesis by preventing FXR inhibition in the ileum (87). Gut bacteria play therefore key roles in bile acid synthesis, composition, and signaling. Conversely, bile acids, in turn, impact and regulate gut microbiota through their antimicrobial effects, suggesting that the relationship between microbiota and bile acids is complex and bidirectional and that a dynamic interplay exists between bile acid pool, and the bacterial population in the gut (88, 89).

It should be noted that the murine bile acid pool is very different than the one found in humans. In mice, the most abundant bile acids are hydrophilic bile acids, muricholic acids, and cholic acid. In humans, bile acid pool is rather hydrophobic and consists mostly of chenodeoxycholic acid and cholic acid (90). These substantial differences in bile acid composition should be considered when using animal models to study the interaction between the gut microbiota and bile acids. The experimenter needs to be attentive to interpretation and translation of mice data that might be poor predictors of human experience (91).

Although the complete signaling capacity of bile acids remains incompletely understood, it is well-established that bile acids, by activating FXR and TGR5, are involved in host glucose homoeostasis and energy metabolism. Notably, FXR is expressed in different metabolically active tissues including in liver, gut, and white adipose tissue (92) and both conjugated and non-conjugated bile acids can interact with FXR, with chenodeoxycholic acid described to be its most potent activator (93) whereas other bile acids are suspected to be FXR antagonists (87).

Importantly, long-term oral treatment with FXR agonist in obese mice leads to exacerbated weight gain and glucose intolerance (94) while obese mice deficient for FXR show improved metabolic parameters. These transgenic mice are characterized by lower body weight gain and improved glucose control as a result of an enhanced glucose clearance and better adipose tissue insulin sensitivity (95, 96). However, hepatic FXR deficiency did not protect animals from diet-induced obesity and insulin resistance, suggesting a role for non-hepatic FXR in the control of glucose homeostasis in obese state (95).

Most publications indicate that activation of intestinal FXR has a negative impact on host glycemic control in the obese state, suggesting that inhibition of intestinal FXR signaling might be efficient for managing hyperglycemia (97, 98).

Paradoxically, some authors have discovered a mechanism in which activation of intestinal FXR with intestine-restricted FXR agonist fexaramine (FEX) markedly modulated the gut microbiome leading to an activation of TGR5/GLP-1 signaling to effectively improve insulin sensitivity and glucose tolerance (99). Furthermore, antibiotic treatment reversed the beneficial metabolic effects of FEX in obese and diabetic mice, suggesting a critical role of gut microbiota. Collectively, studies tend to show that manipulation of FXR leads to complex alterations in glucose homeostasis but further investigation is needed to clarify its exact contribution in host glycemic response.

The expression and production of the entero-hormone FGF19 are also directly controlled by bile acids, and recent reports revealed that besides its well-known action in the feed-back regulation of hepatic bile acid production, FGF19 circulates in the body, and exerts important metabolic actions. For example, FGF19 has been described to stimulate hepatic glycogen synthesis in postprandial state, hence contributing to reduce blood concentrations of glucose (100). In mice, it was also demonstrated that systemic FGF19 administration can dramatically improve glucose tolerance through its action in the brain (101). Finally, recent data from our laboratory highlighted a novel role of FGF19 in the regulation of the muscle mass and reduction of adipose tissue weight (102). Altogether, one can suggest that increasing FGF19 production, via bile acid and/or microbiota modulation could be useful in the design of novel strategies to control diabetes and obesity state.

Interestingly, a growing body of evidence suggests that microbiota-bile acids interactions play also a role in the rapid improvement of glycemic control after bariatric surgery (103), since altered microbiota observed in bariatric patients reduced body fat gain when transplanted into germ free mice (104). Along with a modified gut microbiota, patients who underwent Roux-en-Y gastric bypass surgery presented increased postprandial bile acid and FGF19 response compared with obese control patients without surgery (91, 105).

Although it is complex and not fully understood, bile acid homeostasis is another piece in the puzzle linking microbiota, and host glycemic control. Modulation of the gut microbiota-bile acids axis may offer a future therapeutic approach in obesity and T2DM.

## Adipose Tissue Inflammation and Function

Emerging evidence suggests that microbes resident in the human intestine are involved in host glycemic regulation through adipose tissue regulation (106, 107). Adipose tissue has long been described as an inert tissue with sole function to store lipids. Nowadays, adipose tissue is commonly recognized as an active organ presenting key metabolic and endocrine functions and playing a critical role in host metabolism. The adipose tissue is a pivotal regulator of whole-body energy homeostasis. In excess nutrient conditions, the adipose tissue stores surplus nutrients in the form of neutral lipids, whereas in nutrient deficit conditions, it supplies fuel to other tissues through lipolysis (108). In the process of stocking excess lipids, the aberrant expansion of white adipose mass, via hyperplasia, and adipocyte hypertrophy, leads to cellular stress and local inflammatory response characterized by macrophage infiltration and the release of pro-inflammatory cytokines (109, 110). This chronic low-grade inflammation of the adipose tissue participates in obesity, insulin resistance, hyperglycemia development, and therefore T2DM pathophysiology (111, 112). Although the etiology of this obesity-related inflammatory process remains unclear and probably involves a complex network of signals interconnecting several organs, a growing body of evidence suggests that gut microbiota is one of the players in the development and maintenance of this low-grade systemic inflammation. Cani et al. have described the link between gut bacteria and metabolic inflammation by identifying bacterial LPS as an inflammatory factor causative of the onset of insulin resistance, obesity, and diabetes (113). This phenomenon, known as metabolic endotoxemia, dysregulates the inflammatory tone, and triggers body weight gain and diabetes; overall suggesting that lowering plasma LPS concentration could be a potent strategy for the control of T2DM. A healthy gut microbiota is essential to support the barrier function of the intestinal epithelial barrier by maintaining tight-junction protein structure and suppressing intestinal inflammation. Conversely, in case of disturbances in the equilibrium of the gut microbiota, intestinal permeability, and translocation of LPS-related endotoxemia increase (114, 115). Studies conducted in humans have also reported increased LPS levels and impaired intestinal barrier functions correlating with body mass index and high fat diet (116–118). Importantly, besides adipose tissue, other organs and tissues including liver, skeletal muscle, pancreas but also heart, and blood vessels can be affected by low-grade inflammation, and can consequently impact glucose metabolism (119, 120). While these alterations should not be neglected, we will mainly focus on adipose tissue in the present review. Indeed, a strong and unique metabolic crosstalk exists between the intestine and the peripheral white adipose tissue through compounds and metabolites produced or induced by gut microbiota, to modulate host glycemic response (107). For example, as mentioned before, gut microbiota can induce incretin GLP-1 secretion, which, in turn, has been further shown to have favorable anti-inflammatory effects on adipocytes and macrophage infiltration in adipose tissue in an obese mouse model of diabetes, possibly contributing to the improvement of insulin sensitivity (121).

Several probiotic bacterial strains have been described to play a proactive role in preventing or inhibiting the chronic low-grade inflammation in adipose tissue leading to the attenuation of insulin resistance and hyperglycemia state. A supplementation with probiotic of Indian gut origin *Lactobacillus fermentum* MTCC5689 for a period of 6 months has been shown to reduce pro-inflammatory gene markers TNFα and IL-6 in visceral fat of high fat diet fed mice (122). Consistently, *Lactobacillus sakei* OK67 has been described to ameliorate high-fat diet-induced hyperglycemia and obesity by lowering the inflammatory state and increasing the expression of colon tight junction proteins in mice (123). Authors indeed showed that a treatment of 4 weeks leads to decreased fasting blood glucose levels and glycemic control after an oral glucose tolerance test, along with an inhibition of TNF-α and IL-1β expression induced by high fat diet. Moreover, *Lactobacillus sakei* OK67 treatment induced NF-κB activation in the colon, and significantly increased anti-inflammatory cytokine IL-10 and tight junction proteins expression in the colon.

The white adipose tissue exhibits great plasticity and can undergo “browning” (also called beiging) by acquiring features of brown adipose tissue. This transition takes usually place under certain conditions such as cold exposure or chronic endurance exercise, and transforms white adipose tissue into beige adipose tissue. Beige adipose tissue exhibits higher thermogenesis rate and enhanced capacity to dissipate energy through UCP1-mediated mitochondrial uncoupling (124). Because of their energy-burning capabilities, beige, and brown adipose tissues have become a focus of metabolic diseases research and have led to a high interest in the identification of “browning agents” to combat the development of obesity and associated pathologies (125).

Several studies have indeed highlighted that adipose tissue browning promoted a lean and healthy metabolic phenotype including by improving insulin sensitivity. In 2015, Suarez-Zamorano et al. uncovered a mechanism linking gut microbiota, beige fat development, and metabolic disorders (126). They pointed out that a depletion of the gut microbiota, induced by antibiotic treatment or mice raised in axenic conditions, enhanced the development of functional subcutaneous, and visceral beige adipose tissue. This expansion of beige fat, in turn, promoted an improved glucose control, enhanced insulin sensitivity along with a reduction of white fat and adipocytes size in lean mice and different models of obese mice. The beneficial metabolic profile and the browning of the subcutaneous fat were reversed by recolonization of the mice intestine, confirming the involvement of the gut microbiota in this process.

More recently, Chen et al. have identified that probiotic strain *Lactobacillus reuteri* 263 mediates its anti-obesity effect through energy metabolism remodeling of white adipose tissue in a model of obese rats (127). Authors showed that *Lactobacillus reuteri* 263 enhanced oxygen consumption in white adipose tissue after 8 weeks of daily treatment. The mRNA expressions of thermogenesis genes were up-regulated in the white adipose tissue of treated group compared to the control group. Furthermore, *Lactobacillus reuteri* 263 induced browning of white adipose tissue due to an upregulation of browning-related genes mRNA levels. The analysis of a human cohort has reinforced data showing a link between gut bacteria and adipose tissue browning; 34 morbidly obese women and men have been analyzed in order to identify a potential relationship among gene expression markers of adipose tissue browning and gut microbiota composition (128). Authors conclude that *Firmicutes* relative abundance is positively associated with markers of brown adipose adipocytes in subcutaneous adipose tissue and insulin sensitivity. Geurts et al. provided evidence that the endocannabinoid system might also have a role in the interplay between gut microbiota, adipose tissue browning, and glucose tolerance (129). Several gut microbiota-derived compounds have been described to link intestine and regulation of the host energy metabolism through adipose tissue remodeling. For example, KetoA, a linoleic acid metabolite produced by gut lactic acid bacteria, has been shown to up-regulate genes involved in brown adipocyte functions, including UCP1, in white adipose tissue further enhancing energy expenditure in mice and thereby protecting them from diet-induced obesity and improving overall metabolic health (130).

In connection with previous section, bile acids have been described to activate thermogenesis and adipose tissue browning, therefore improve metabolic control (131). The administration of bile acids to mice has been shown to enhance energy expenditure in brown adipose tissue leading to obesity and resistance to insulin prevention. These effects have been suggested to be mediated by the receptor TGR5 (131). In line with these data, a treatment with chenodeoxycholic acid for 8 weeks in obese mice has been shown reverse obesity via induction of UCP1 in brown adipose tissue (132).

Numerous compounds, such as gypenosides from jiaogulan tea or Danshensu Bingpian Zhi, a synthetic derivative of natural compounds used in traditional Chinese medicine, have been lately described to modulate both gut microbiota composition and adipose thermogenesis leading to a better host glycemic response (133, 134). Notably, beneficial effects of gypenosides have been associated with decreased ratio of *Firmicutes* to *Bacteroidetes* and increased *Akkermansia muciniphila* abundance in the gut microbiota, together with and increased amount of brown adipocytes in high fat diet-fed mice (133). These compounds are therefore seen as beneficial prebiotic agents improving obesity-related metabolic syndrome, including insulin resistance, and inflammation in obese individuals, through adipose tissue browning and modulation of intestinal microbiota composition.

## Conclusions

The gut microbiota has become the subject of considerable investigation in recent years and our knowledge of its composition and functions is rapidly growing.

It is becoming increasingly clear that gut microbiota has profound impact on host physiological and pathophysiological processes. Notably, it is now well-established that imbalanced gut microbiota is linked to host glycemic control impairment and T2DM development, as showed by the findings we have reviewed.

Although the precise role of gut microbiota remains incompletely understood, as suggested by multiple studies that remain controversial, several key molecular mechanisms linking gut bacterial residents, and host glucose homeostasis have been highlighted and include: intestinal incretin secretion, SCFA production, bile acid metabolism, and adipose tissue regulation. Some of these bacterial processes can be targeted to improve the treatment of T2DM patients, suggesting that these approaches appear as a promising avenue for the treatment of glycemic disorders. As a matter of fact, despite a large therapeutic arsenal, the treatment of this complex chronic disease is far from being optimal since in a majority of patients, T2DM remains poorly controlled over time. Using pre/probiotics in order to modulate blood glucose has been considered for a long time, and the discovery of the key roles of gut bacteria in T2DM has boosted research efforts in this field. However, the low efficacy of currently available pre/probiotic strategies highlights the lack of well-defined and standardized methodology to identify and select compounds and bacterial strains with proven antidiabetic properties. This is probably the challenge of the next 5 years. A better understanding of the mechanisms by which gut microbiota impacts host physiology will help defining innovative and efficient strategies to identify probiotic strains with antidiabetic activities or nutritional interventions increasing their abundance in the gut. Although preclinical models will probably result in the rapid identification of new and efficient probiotic strains, the transfer of these data to the human situation will require large and well performed clinical trials in the next few years. Therefore, a deeper understanding of the tight interplay between gut microbiota and host glycemic control and a validation of this knowledge in humans are still needed to optimize and individualize gut microbiota manipulation to combat metabolic diseases and improve overall host metabolic health.

## Author Contributions

All authors listed have made a substantial, direct and intellectual contribution to the work, and approved it for publication.

### Conflict of Interest Statement

The authors declare that the research was conducted in the absence of any commercial or financial relationships that could be construed as a potential conflict of interest.
